# Host cell-based screening assays for identification of molecules targeting *Pseudomonas aeruginosa* cyclic di-GMP signaling and biofilm formation

**DOI:** 10.3389/fmicb.2023.1279922

**Published:** 2023-11-15

**Authors:** Ying Hu, Jeremy Stephen Webb, Shi-qi An

**Affiliations:** National Biofilms Innovation Center and School of Biological Sciences, University of Southampton, Southampton, United Kingdom

**Keywords:** antibiotic resistance, c-di-GMP, biofilm formation, Pseudomonas aeruginosa, drug screening

## Abstract

The rapid emergence of bacterial resistance to antibiotics in current use is occurring worldwide and poses a significant threat to global healthcare systems. Recent research to identify new effective anti-bacterial agents has focused on regulatory pathways as targets for interference. Regulatory mechanisms employing intracellular Bis-(3′,5′) cyclic di-guanylate (c-di-GMP) as a secondary messenger represent a distinct category of subjects. This molecule, c-di-GMP, is present in nearly all bacterial species and plays a pivotal role in governing various biological processes, encompassing antibiotic resistance, biofilm formation, and virulence. Alteration of the cellular concentrations of the nucleotide through modulation of associated signaling pathways has the potential to reduce biofilm formation or increase susceptibility of the biofilm bacteria to antibiotics. Here, we have developed a screen for compounds that alter c-di-GMP levels in *Pseudomonas aeruginosa* in co-culture with bronchial epithelial cells. Through the assay of 200 natural compounds, we were able to identify several substances showing promising effects on *P. aeruginosa* in a host biofilm infection model. Importantly, we detected compounds that inhibit c-di-GMP levels and showed significant influence on biofilm formation and virulence in *P. aeruginosa in vitro* and *in vivo*. Consequently, we offer proof-of-concept information regarding swift and practical drug screening assays, suitable for medium- to high-throughput applications, which target the c-di-GMP signaling pathways in this significant Gram-negative pathogen.

## Introduction

Antibiotic-resistant bacterial infections pose a significant global health challenge, demanding the development of novel antimicrobial agents (Caly et al., 2015; Andersen et al., 2021). Traditional antibiotics are becoming less effective in eradicating these infections, underscoring the urgent need for alternative approaches (Caly et al., 2015; Andersen et al., 2021). Recent research efforts have focused on targeting regulatory pathways, with the intracellular second messenger c-di-GMP emerging as a promising drug target (Caly et al., 2015; Andersen et al., 2021). C-di-GMP serves as a signaling molecule present in virtually all bacteria, regulating crucial processes including antibiotic resistance, biofilm formation, and virulence (Römling, 2023).

*Pseudomonas aeruginosa*, an opportunistic human pathogen, is of particular concern due to its association with acute and chronic infections in immunocompromised and hospitalized patients (Caly et al., 2015; Andersen et al., 2021). It is notorious for causing infections in individuals with conditions such as cystic fibrosis, severe burns, chronic wounds, chronic obstructive pulmonary disease, and those with implanted biomaterials. The ability of *P. aeruginosa* to form biofilms presents a significant challenge, rendering the bacteria highly resistant to antibiotics and host immune defenses (An et al., 2019).

Targeting the c-di-GMP signaling pathway in *P. aeruginosa* offers a promising strategy to combat antibiotic-resistant infections (Caly et al., 2015; An and Ryan, 2016; Andersen et al., 2021). By manipulating c-di-GMP levels, it is possible to influence key virulence traits of the bacterium, including biofilm formation and the production of virulence factors (Bjarnsholt et al., 2010; Andersen et al., 2021; Mamat et al., 2023; Römling, 2023). This approach provides a unique opportunity to attenuate the pathogenicity of *P. aeruginosa* without exerting selective pressure for the development of antibiotic resistance.

In this study, we aimed to identify compounds capable of modulating c-di-GMP levels and inhibiting *P. aeruginosa* host associated biofilm formation and virulence. To do this, we developed and employed a screening model that involved co-culturing human airway epithelial cells with *P. aeruginosa*, creating a physiologically relevant environment for biofilm formation. By screening a diverse set of natural products, known for their chemical diversity and biological activities, we identified lead compounds that can effectively target c-di-GMP signaling and disrupt virulence and biofilm formation in *P. aeruginosa*. Of note, we could identify several novel chemical entities that were able to protect host cells from *P. aeruginosa* biofilm formation and cell death. One compound, 6,7-dihydroxycoumarin, was capable of modulating c-di-GMP signaling in *P. aeruginosa*. This compound exhibited the ability to reduce cytotoxicity, inhibit biofilm formation in an *in vitro* co-culture biofilm model, and decrease mortality in an *in vivo Galleria mellonella* infection model. The data furnish proof-of-concept evidence for fast and feasible medium- to high-throughput screening assays for novel compounds, aimed at modulating c-di-GMP levels. Such endeavors hold promise in facilitating the discovery of potent anti-biofilm agents.

## Materials and methods

### Bacterial strains, plasmids, and growth conditions

The strains and plasmids used in this study are listed in Supplementary Table 1. For routine strain manipulations, *P. aeruginosa* strains were routinely grown at 37°C, in Luria broth (LB) with shaking or on 1.5% LB agar plates. When appropriate, the following concentrations of antibiotics were used: for *P. aeruginosa* strains, gentamicin (Gm) at 50 μg/mL, streptomycin (Str) at 200 μg/mL, tetracycline (Tc) at 100 μg/mL; for *Escherichia coli* strains, ampicillin (Amp) at 100 μg/mL, 6 μg/mL at chloramphenicol (Cm) 6 μg/mL, kanamycin (Km) at 35 μg/mL, and gentamicin (Gm) at 15 μg/mL. Antibiotics were purchased from Sigma-Aldrich.

### Human bronchial epithelial cell culture

Human bronchial epithelial cell line 16HBE 14o- was a gift from Professor Jane Lucas (University of Southampton, United Kingdom) and was cultured in Gibco™ Minimum Essential Media (Merck Group) supplement with 10% fetal bovine serum (FBS; Merck Group), 2 mM L-glutamine (Merck Group), and 50 U/mL Gibco™ Penicillin–streptomycin (Thermos Fisher Scientific) at 37°C with 5% CO_2_. The medium was changed on the second day. The cells were passaged every 4–5 days (80% confluence).

Minimum Essential Medium (MEM) Eagle (Thermo Fisher Scientific) supplemented with 2 mM L-glutamine and 0.4% arginine (Merck Group) was used in the co-culture biofilm model, and incubated at 37°C and 5% CO_2_, as previously described (Twomey et al., 2011).

### Sources of the screened compounds

The Puretitre natural compound library purchased from Caithness Biotechnologies, United Kingdom was used in this work.^1^ The library contains a collection of 200 compounds found in natural products used as traditional medicines, or as the basis of modern drugs. The follow-up compounds used after the primary screen were also purchased from Caithness Biotechnologies, United Kingdom. All of the compounds were maintained in DMSO.

### Construction of the dual-labeled reporter strain PA14-*lux* (pCdrA::*gfp^C^*)

The growth and c-di-GMP levels in *P. aeruginosa* in different conditions were assessed using a *lux* reporter and a *gfp* reporter, respectively. In order to create the dual-labeled reporter strain, WT PA14 was first tagged with the mini-Tn7 reporter construct from pUC18T-mini-Tn7T-*lux*-Gm by means of four-parental mating with *E. coli* DH5α harboring the delivery vectors as donor strains and with *E. coli* strains harboring pUX-BF13 and pRK600 as helper strains, as previously described (Damron et al., 2013). Then, the plasmid-based reporter pCdrA::*gfp^C^* (Rybtke et al., 2012) was introduced into the *lux*-labeled background strains by electroporation. The reporter’s activity was tested using BMG CLARIOstar multi-mode microplate reader.

### General screening procedure

**Figure 1:**
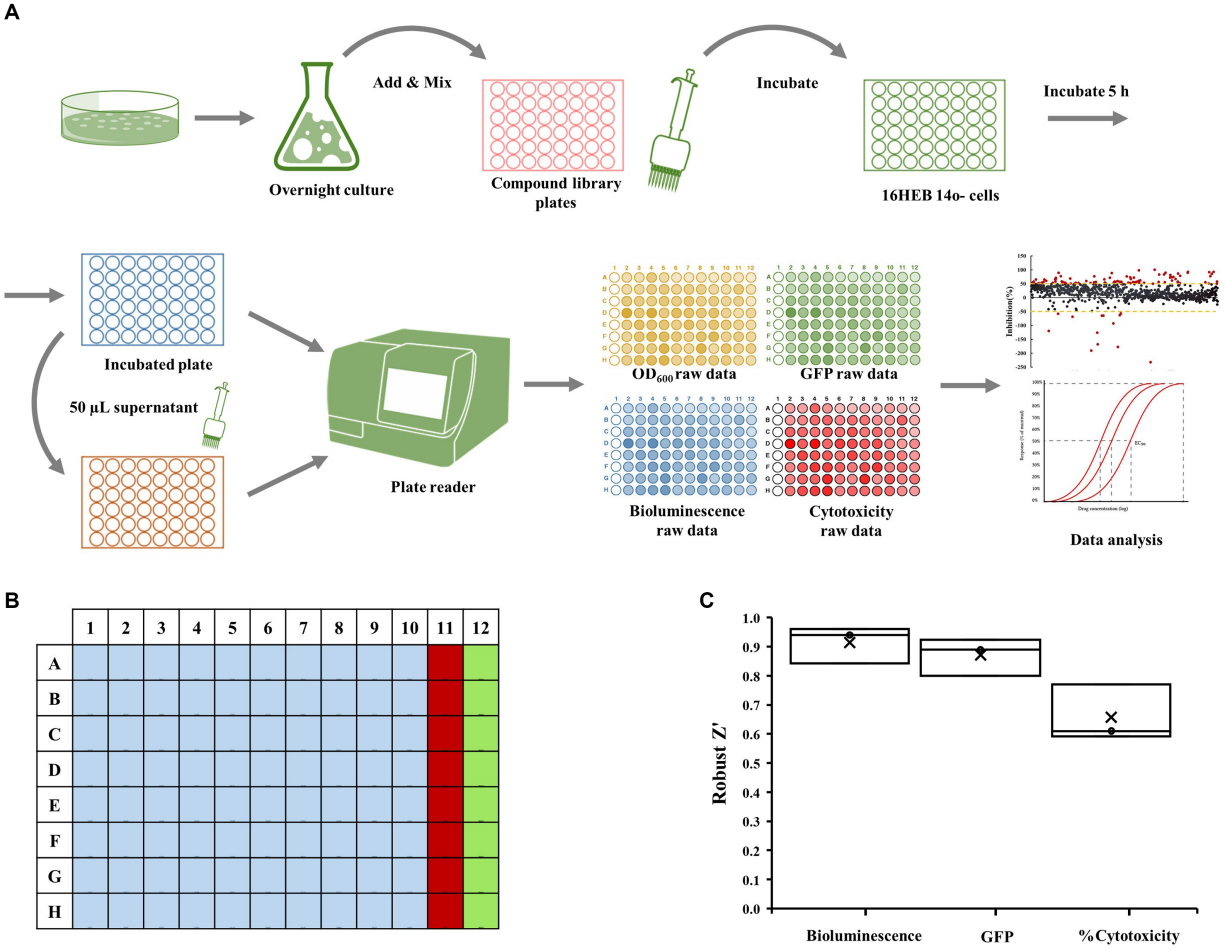
Epithelial cell coculture-screening assay. **(A)** Schematic representation of co-culture screening assay. The dual-labeled reporter strain was cultured overnight in LB medium. Bacterial cells were collected and suspended before being added to 96-well plate along with compounds from the Puretitre natural compound library. The mixture was then transferred to 96-well plate containing 16HBE14o-Human Bronchial Epithelial Cells. After a 5-h incubation at 37°C with 5% CO_2_, aliquots of supernatant were collected for cytotoxicity analysis, and the bioluminescent and GFP values were measured using a multi-mode microplate reader. **(B)** A 96-well plate map used for screening assay. Well A1-H10 (light blue) contain the compounds from the library. The “positive control” (red) consisting of a final concentration of 50 μg/mL tobramycin sulfate is added to wells A11-H11 and the “negative control” (green) containing a final concentration of 1% DMSO is added to wells A12-H12. (**C)** The robust *z*’ values for bioluminescent, cytotoxicity, and GFP are calculated according to the formula provided in the section “Materials and methods.”

The reporter strain PA14-*lux* (pCdrA::*gfp^C^*) was grown overnight in LB medium. Bacterial cells were collected and suspended in the MEM Eagle medium to achieve an optical density at 600 nm of 0.01. The diluted culture was then added to 96-well plates containing the compounds from Puretitre natural compound library (final con. 250 μM). After thorough mixing, the mixture is transferred to 96-well plate containing 16HBE14o-Human Bronchial Epithelial Cells (with supernatant removed). Following a 4.5-h incubation 37°C with 5% CO_2_. 50 μL of supernatant is collected for cytotoxicity analysis, while bioluminescent (Emission max 580-80 nm), and GFP (Excitation max 560 nm, Emission max 590 nm) values are measured using BMG CLARIOstar multi-mode microplate reader. Wells A23–P23 were designated as the “positive control” and contained a final concentration of 50 μg/mL tobramycin sulfate. Wells A24–P24 served as the “negative control” and contained a final concentration of 2.5% DMSO (Figure 1).

### Cytotoxicity assays

Cytotoxicity was determined by evaluating the activity levels of lactate dehydrogenase (LDH) in the medium with Invitrogen™ CyQUANT™ LDH Cytotoxicity Assay (fluorescence) kit according to the manufacturer’s instructions. Briefly, aliquots of supernatant from the compound library-screening assay and co-culture biofilm assay were taken and added to the reaction mixture from the kit. After incubation at room temperature, the reaction was stopped by adding the stop solution from the kit, and the fluorescence was measured with a plate reader by using an excitation of 560 nm and an emission of 590 nm. %Cytotoxicity was calculated by using the following formula:


$$\%\mathrm{Cytotoxicity}=\left[\frac{\begin{array}{l} \mathrm{Compound}-\mathrm{treated}\;LDH\;\mathrm{activity}-\\ \mathrm{Spontaneous}\;LDH\;\mathrm{activity} \end{array}}{\begin{array}{l} \mathrm{Maximum}\;LDH\;\mathrm{activity}-\\ \mathrm{Spontaneous}\;LDH\;\mathrm{activity} \end{array}}\right]\times 100$$

### Data analysis

Evaluate the uniformity and reproducibility of the assay using robust *z*’ analysis, which is the most common quality metric reported for high-throughput screens (Rugjee et al., 2016). Calculate robust *z*’ using the formula:


$$\mathrm{robust}\;z^{\prime }=1-\frac{3\left({\mathrm{MAD}}_{n}+{\mathrm{MAD}}_{p}\right)}{\left|{\mathrm{median}}_{n}-{\mathrm{median}}_{p}\right|}$$

Where Median Absolute Deviation (MAD) is an estimate of standard deviation, *p* is the positive control and *n* is the negative control. An assay with a robust *z*’. An assay with a robust *z*’ value (calculated for bioluminescent, GFP raw data, and %Cytotoxicity) greater than or equal to 0.5 is considered an excellent assay.

To calculate %inhibition of the intracellular level of c-di-GMP using the formula:


$${\mathrm{Inhibition}}_{GFP}=100\%\times 1-\frac{\begin{array}{l} ({\mathrm{Xi}}_{GFP}-{\mathrm{median}}_{p\left(GFP\right)}/\\ \left({\mathrm{Xi}}_{\mathrm{lux}}-{\mathrm{median}}_{p\left(\mathrm{lux}\right)}\right) \end{array}}{\begin{array}{l} \left({\mathrm{median}}_{n\left(GFP\right)}-{\mathrm{median}}_{p\left(GFP\right)}\right)/\\ \left({\mathrm{median}}_{n\left(\mathrm{lux}\right)}-{\mathrm{median}}_{p\left(\mathrm{lux}\right)}\right) \end{array}}$$

Where Xi_GFP_ is the iterated GFP value of each sample well. %Inhibition_GFP_ > 0, c-di-GMP inhibitor; and %Inhibition_GFP_ < 0, c-di-GMP promoter.

To calculate %inhibition of cytotoxicity using the formula:


$$\%{\mathrm{Inhibition}}_{\mathrm{Cytotoxicity}}=100\%\times 1-\frac{\left(\begin{array}{l} {\mathrm{Xi}}_{\%\mathrm{Cytotoxicity}}-\\ {\mathrm{median}}_{p\left(\%\mathrm{Cytotoxicity}\right)} \end{array}\right)}{\begin{array}{l} |{\mathrm{median}}_{n\left(\%\mathrm{Cytotoxicity}\right)}-\\ {\mathrm{median}}_{p\left(\%\mathrm{Cytotoxicity}\right)}| \end{array}}$$

Where Xi_%cytotoxicity_ is the %Cytotoxicity value of each sample well. %Inhibition_Cytotoxicity_ > 0, cytotoxicity inhibitor; %Inhibition_Cytotoxicity_ < 0, cytotoxicity promoter.

#### Dose–response curve

The selected hit compound(s) was further tested by a 10-point dose–response assay with a top concentration of 2 mM and 2-fold dilution series. All experiments were performed three times. Results are expressed as mean value ± standard deviation (SD). IC_50_ calculations were performed using the built-in algorithms for dose–response curves with variable slope using GraphPad Prism software (version 9.5.1).

#### Co-culture biofilm assay

The co-culture biofilm formation assay used in this study is based on the ability of *P. aeruginosa* to adhere and form biofilms on human airway epithelial cells grown in culture, as previously described (Klausen et al., 2003; Moreau-Marquis et al., 2010). 16HBE14o- cells were seeded in μ-Slide 8 Well Chamber Slide (ibidi, Thistle Scientific). The cells were grown to form a confluent monolayer before being inoculated with GFP-tagged, *P. aeruginosa* WT PA14 strain alone (~ 2 × 10^6^ CFU), or in combination with tobramycin (final concentration: 1000 μg/mL), the test compound 6,7-Dihydroxycoumarin (final concentration: 207 μM), or 6,7-Dihydroxycoumarin plus tobramycin. After incubating the slides for 1 h at 37°C with 5% CO_2_, the supernatant was removed and replaced with fresh medium supplemented with 0.4% arginine. The slides were then further incubated at 37°C with 5% CO_2_ for 5 h. The integrity of the airway epithelial cells and the development of GFP-labeled *P. aeruginosa* biofilms at the apical surface of airway epithelial cells were visualized by confocal microscopy.

#### Confocal laser scanning microscopy

The integrity of the airway epithelial cells and the development of GFP-labeled *P. aeruginosa* biofilms were visualized by Leica (SP8) confocal microscope equipped with a 63X oil objective (Imaging and Microscopy Center, University of Southampton). The 16HBE cells were pre-stained with CellBrite™ Orange Cytoplasmic Membrane Dyes (excitation/emission: 549/565 nm, Cambridge Bioscience). Biofilms formed by GFP-tagged PA14 strain were observed by monitoring the GFP fluorescence. Image data were processed with Leica LAS AF software (Imaging and Microscopy Center, University of Southampton), and quantitative analysis was performed using IMARIS software (Bitplane, Oxford Imaging, United Kingdom). Each three-dimensional (3D) image was created from 15 *z*-stacks, captured at 1 μm intervals, resulting in a 15 μm imaging depth. At least four *z*-stack images from each of three independent experiments were used for each analysis.

#### *Galleria mellonella* infection assays

*Galleria mellonella* were purchased from Blades Biological Ltd., United Kingdom and stored in wood chippings at 15°C in darkness to prevent pupation. Larvae weighing 300 ± 30 mg with no cuticle discoloration were selected. Ten healthy larvae per treatment and controls were used per experimental parameter. All experiments were performed independently on three separate occasions. In the pathogenicity studies, 10 larvae were inoculated through the last left pro-leg into the hemocoel using a 25 μL syringe (Merck Group) with 10 μL of PBS-washed WT PA14 culture (approximately 1 × 10^3^ CFU/mL). 1 h after inoculation, larvae were treated with either tobramycin (final concentration: 1 mg/Kg), the test compound 6,7-Dihydroxycoumarin (final concentration: 200 mg/Kg), or the compound plus tobramycin. Larvae inoculated with PBS were utilized as controls. The injected larvae were placed in Petri dishes containing filter paper and were incubated at 37°C. Mortality, cuticle discoloration, and response to touch were recorded 16 h post-treatment. All experiments were performed independently on three separate occasions.

## Results

### Construction of a viability and c-di-GMP-responsive *Pseudomonas aeruginosa* dual-labeled reporter strain

In order to identify compounds that potentially modulate the c-di-GMP signaling during infection *P. aeruginosa* reference strain PA14, we established a co-culture-based screening assay. We constructed a dual-labeled reporter strain that allowed the library of natural compounds to be screened for their effects on both modulation of bacterial c-di-GMP signaling and growth during infection. The WT PA14 strain was initially marked using the mini-Tn7 reporter construct obtained from pUC18T-mini-Tn7T-lux-Gm through a four-parental mating approach. This involved *E. coli* DH5α strains carrying the delivery vectors as donor strains and *E. coli* strains containing pUX-BF13 and pRK600 as helper strains, as previously outlined in Damron et al. (2013). Then, the plasmid-based reporter pCdrA::*gfp^C^* (Rybtke et al., 2012) was introduced into the lux-labeled background strains by electroporation, generating the dual-labeled reporter strain named PA14-lux (pCdrA::*gfp^C^*; Supplementary Table 1). The experimental strain PA14-*lux* (pCdrA::*gfp^C^*) and the control strain WT PA14 were cultivated in Luria-Bertani (LB) medium. Bioluminescence and GFP fluorescence were measured using a multimode microplate reader. Supplementary Figure 1A illustrates that WT PA14 exhibited background-level bioluminescence, whereas PA14-*lux* (pCdrA::*gfp^C^*) displayed an elevated bioluminescent signal. As seen in Supplementary Figure 1B, the baseline reference is set by the GFP expression of the WT PA14 strain, represented as 100%. Without antibiotic treatment, the dual-labeled reporter strain PA14-*lux* (pCdrA::*gfp^C^*) exhibits similar GFP expression to the WT strain due to low c-di-GMP levels, resulting in low cdrA::*gfp* transcription. However, the addition of Ampicillin (100 μg/mL) and Gentamicin (5 μg/mL) induces a significant increase in GFP expression in PA14-*lux* (pCdrA::*gfp^C^*), surpassing the WT strain by over 20%. Sub-minimal inhibitory concentration (sub-MIC) antibiotic exposure has been shown to enhance biofilm formation, elevate c-di-GMP levels, and subsequently promote increased cdrA::*gfp* transcription through heightened c-di-GMP levels within the bacteria (Nolan and Behrends, 2021). These findings suggest that the reporter can serve as a valuable tool for tracking both the viability of *P. aeruginosa* strains and the alterations in c-di-GMP levels induced by drug treatments.

### Human bronchial epithelial cell-based co-culture assay development and validation

The human bronchial epithelial cell-based co-culture with *P. aeruginosa* was used to screen small molecules for inhibition of virulence (measured as loss of cytotoxicity to human cells), modulation of the cellular c-di-GMP level, and inhibition of bacterial growth (Figure 1A). Bacteria were inoculated into plates seeded with 16HBE cells together with test compounds, tobramycin (a positive control) or DMSO (a negative control), according to the layout shown in Figure 1B. Assay quality is measured by Robust *Z*’. The Robust *Z*’ values for bioluminescent, red cytotoxicity, and GFP are calculated according to the formula provided in section Materials and methods (Figure 1C; Here only positive/negative controls are used for Robust Z’ calculation—three replicates). As they are all above 0.5 (average 0.96, 0.87, and 0.66 respectively; Figure 1C), the assay quality was deemed robust.

### Initial screening to identify compounds that modulate the c-di-GMP level during infection

**Figure 2:**
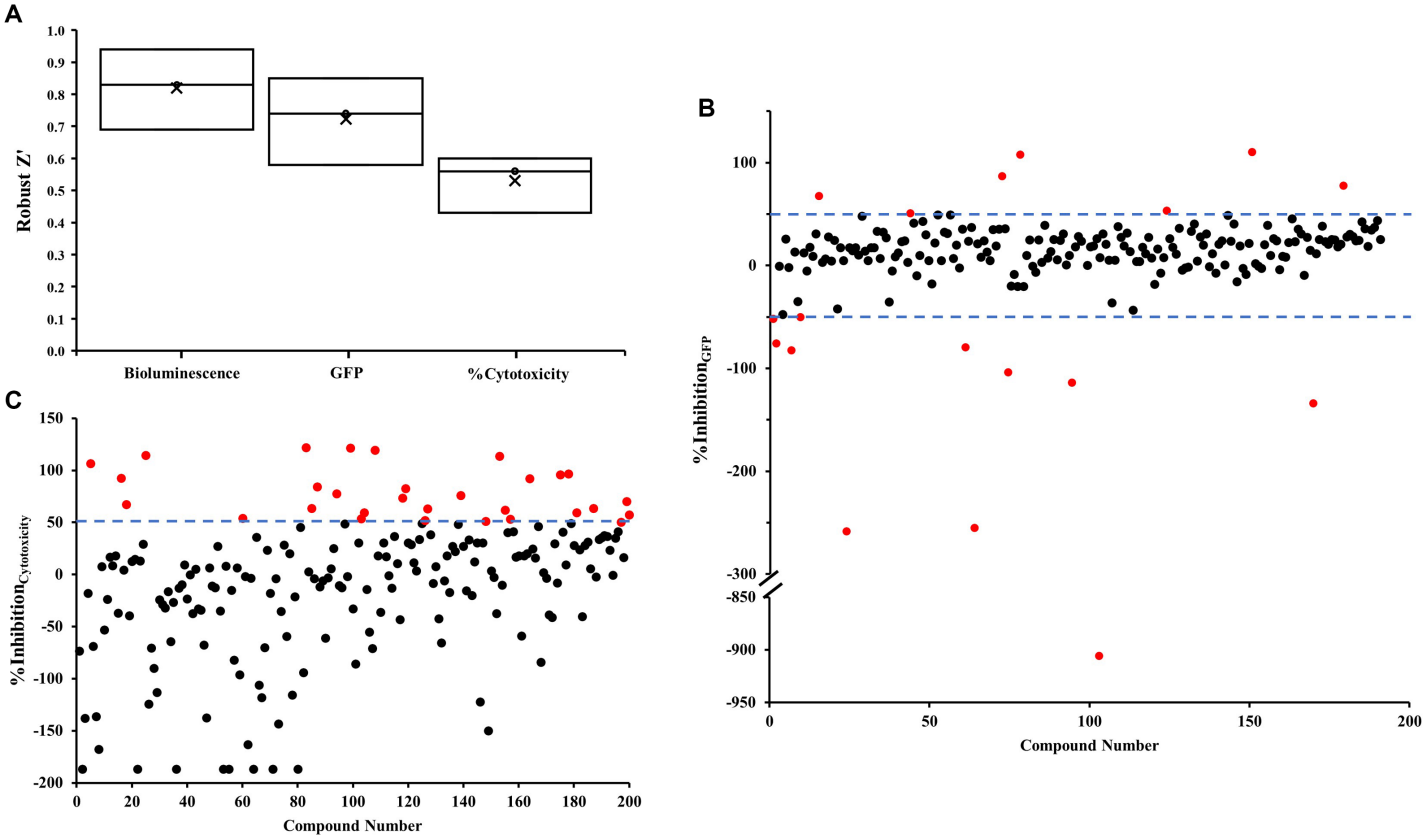
Robust *z*’ and scatter plots for the co-culture screen. **(A)** Robust *z*’ value. **(B,C)** Scatter plots were obtained from each tested small molecule. Each small molecule is represented by a dot and % inhibition distribution for each of the GFP **(B)** and % cytotoxicity **(C)** read-outs are shown. A ± 50% cut-off was selected for hit identification with an effect on GFP and a + 50% cut-off was selected for % cytotoxicity. Potential hits are highlighted in red.

We performed an initial screening of 200 compounds sourced from the Puretitre natural compound library (Caithness Biotechnologies) at a fixed concentration of 250 μM. Representative bioluminescent, GFP raw data, and %Cytotoxicity are shown in Supplementary Figures 2Ai–iii, respectively. A color gradient heatmap has been used to represent well values, with warmer colors like red indicating lower bioluminescent/GFP/red fluorescent values, and cooler colors like green signifying higher values. To assess the efficacy of our assay, we compared the performance of positive controls and negative controls. Robust *z*’ values were calculated to determine the assay’s response window for bioluminescent, GFP, and %Cytotoxicity measurements. The average values obtained were 0.82, 0.72, and 0.53, respectively (Figure 2A). These values demonstrate the reliability and consistency of the screening procedure. Based on the robust quality of the assay, we proceeded to further analyze the acquired data. The % inhibition of cytotoxicity and intracellular c-di-GMP levels (GFP) are determined using the formulas detailed in the section Materials and methods. Representative data for these calculations are presented in Supplementary Figures 2Bi,ii respectively. Well values have been visualized using a color gradient heatmap, where warmer colors such as red represent lower % inhibition, and cooler colors like green signify higher % inhibition. Furthermore, scatter plots illustrating these values from individual wells can be found in Figures 2B,C.

The assay revealed two distinct categories of compounds of interest. Compounds highlighted in Figure 2B appear to possess the potential to regulate intracellular levels of c-di-GMP with minimal effects on bacterial growth, whereas those identified in Figure 2C demonstrate inhibitory properties against cytotoxicity.

To identify compounds with a significant effect on intracellular levels of c-di-GMP, we selected a ± 50% cut-off (indicated by dotted lines) in Figure 2B. Hits that meet this cut-off criterion are denoted by red dots, and a total of 18 compounds were identified in Supplementary Table 2. Out of these, 11 compounds promoted c-di-GMP levels, while seven compounds inhibited them. Notably, we discovered three antibiotics that can regulate c-di-GMP levels but may promote cytotoxicity under the tested conditions.

For compounds with a significant ability to inhibit cytotoxicity, we employed a + 50% cut-off (indicated by dotted lines) in Figure 2C. We identified 30 compounds meeting this criterion (Supplementary Table 3). By integrating the two sets of data, we found that five compounds influenced c-di-GMP levels and inhibited virulence without impacting growth. Among these, four compounds promoted c-di-GMP levels, potentially leading to increased biofilm formation. The remaining compound, 6,7-dihydroxycoumarin (esculetin), inhibited both c-di-GMP levels and cytotoxicity.

### Validation via dose–response assays

**Figure 3:**
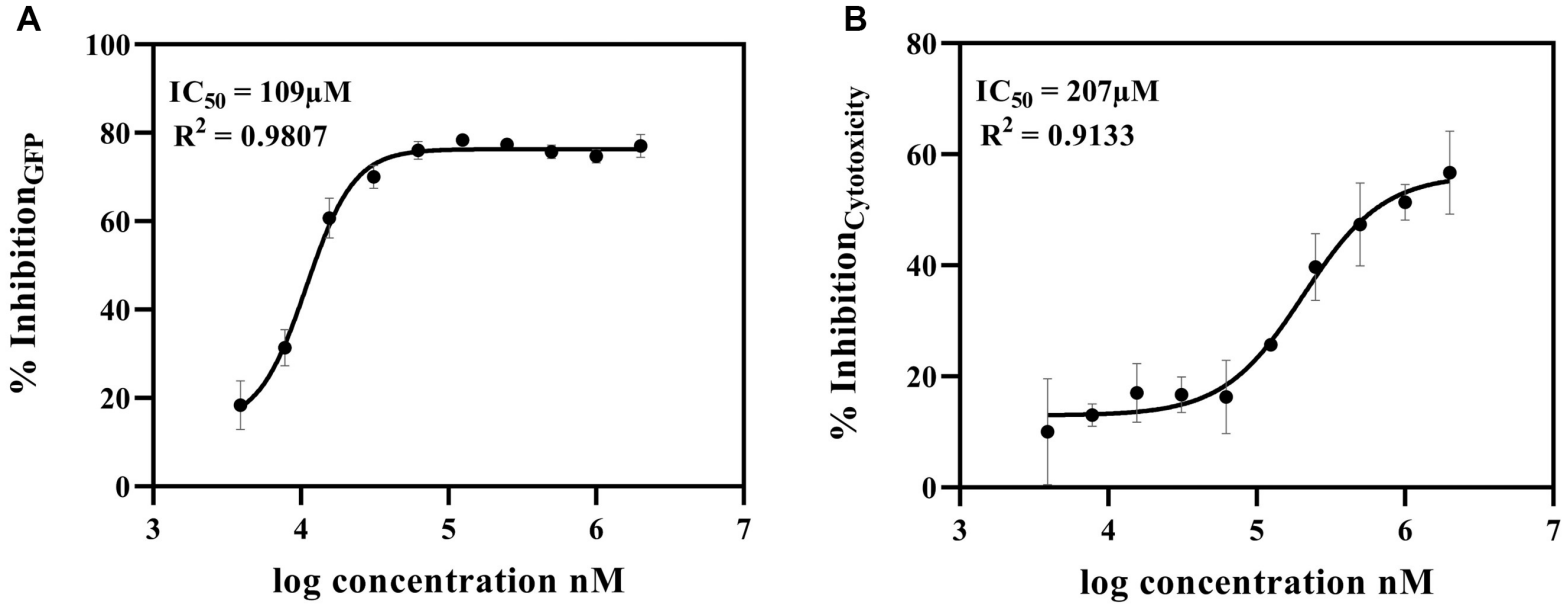
Dose–Response Assay of 6,7-dihydroxycoumarin (esculetin) selected from co-culture human airway epithelial cell-based screen. Potential c-di-GMP **(A)** and cytotoxicity **(B)** inhibitor 6,7-dihydroxycoumarin (esculetin) were further tested in a 10-point dose–response assay with a top concentration of 2 mM and 2-fold dilution series. Fitting a four-parameter logistic function to the data, the IC50 values for inhibition for GFP were 109 μM, and for cytotoxicity was 207 μM.

Selected hit 6,7-dihydroxycoumarin was further tested by 10-point dose–response analysis with a top concentration of 2 mM and a 2-fold dilution series. IC_50_ values were calculated from dose–response curves for 6,7-dihydroxycoumarin as 109 μM for inhibition of GFP levels and 207 μM for inhibition of cytotoxicity (Figures 3A,B, respectively). In addition, we examined whether 6,7-Dihydroxycoumarin could directly influence human cells using the LDH cytotoxicity test to estimate cytotoxicity. The results showed that 6,7-Dihydroxycoumarin alone had little effect on the toxicity of the cells with a percentage reduction in LDH level of 2.1% (Supplementary Figure 3A).

### 6,7-dihydroxycoumarin (esculetin) improves antibiotic efficacy

We investigated whether 6,7-dihydroxycoumarin disrupts biofilm formation using a co-culture system where *P. aeruginosa* biofilms were permitted to develop on epithelial cells. 16HBE14o- cells were seeded in μ-Slide 8 Well Chamber Slide (ibidi, Thistle Scientific). The cells are grown to form a confluent monolayer before being inoculated with GFP-tagged, *P. aeruginosa* WT PA14 strain alone (~ 2 × 10^6^ CFU), or in combination with tobramycin (final concentration: 1,000 μg/mL), the test compound 6,7-dihydroxycoumarin (final concentration: 207 μM), or 6,7-dihydroxycoumarin plus tobramycin (TB). The integrity of the airway epithelial cells and the development of GFP-labeled *P. aeruginosa* biofilms at the apical surface of airway epithelial cells were visualized by confocal microscopy, and biofilm biomass was quantity by the Imaris software (see the section Materials and methods).

**Figure 4:**
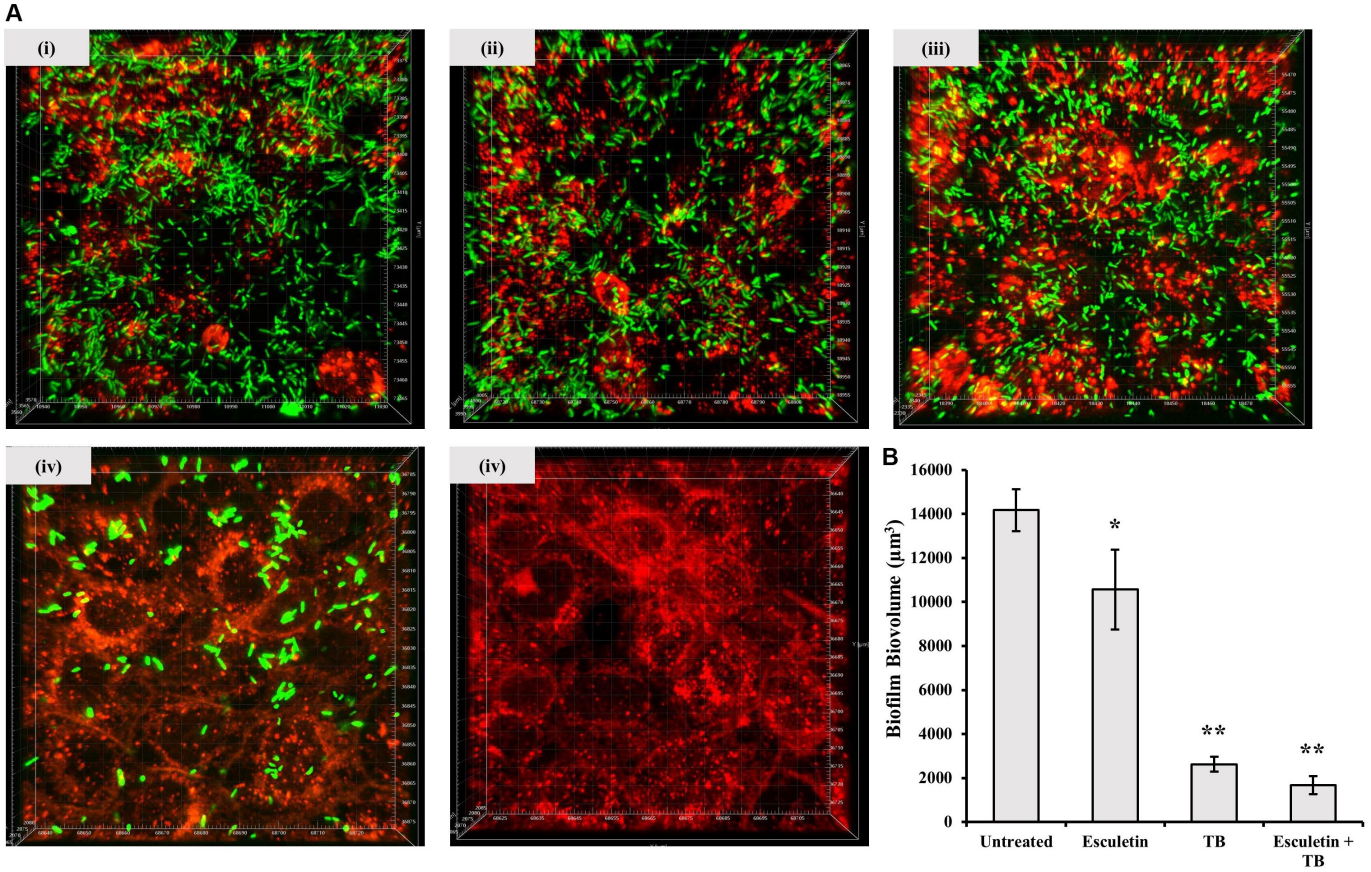
Effects of 6,7-Dihydroxycoumarin on *Pseudomonas aeruginosa* co-culture biofilm. 16HBE14o-cells were seed in μ-Slide 8 Well Chamber Slide then inoculated with GFP-tagged *P. aeruginosa* WT PA14 strain alone **(i)** or in combination with 6,7-Dihydroxycoumarin **(ii)**, tobramycin **(iii)** or 6,7-Dihydroxycoumarin plus tobramycin **(iv)**, cell culture alone without infection were the negative control **(v)**. The 16HBE cells were pre-stained with CellBrite™ Orange Cytoplasmic Membrane Dyes (Red). Biofilms formed by GFP-tagged PA14 strain were observed by monitoring the GFP fluorescence (Green). **(A)** Representative confocal images of biofilm formation. **(B)** Biovolume quantitative analyses were performed on at least four image stacks from three independent experiments. ^*^p < 0.05; ^**^p < 0.01, Student’s *t*-test. Means and standard deviations from triplicate experiments are shown.

As expected, the addition of tobramycin (TB) resulted in a significant reduction in biofilm biomass. When 6,7-dihydroxycoumarin was added alone, it also caused a reduction in biofilm development (Figure 4), although not to the same extent as in the presence of tobramycin (TB). However, at a concentration of 207 μM, 6,7-dihydroxycoumarin further decreased biofilm formation in the presence of tobramycin (Figure 4). In the μ-chambers, 16HBE14o- cells treated with the wild-type strain exhibited compromised membrane integrity, leaving large empty spaces (Figure 4). In contrast, cells treated with 6,7-dihydroxycoumarin in conjunction with tobramycin (TB) maintained their morphology. These results align with the cytotoxicity data, as cells treated with the wild-type strain exhibited high lactate dehydrogenase (LDH) levels, while cells treated with 6,7-dihydroxycoumarin in conjunction with tobramycin (TB) showed a significant reduction in LDH levels (Supplementary Figure 3B).

**Figure 5:**
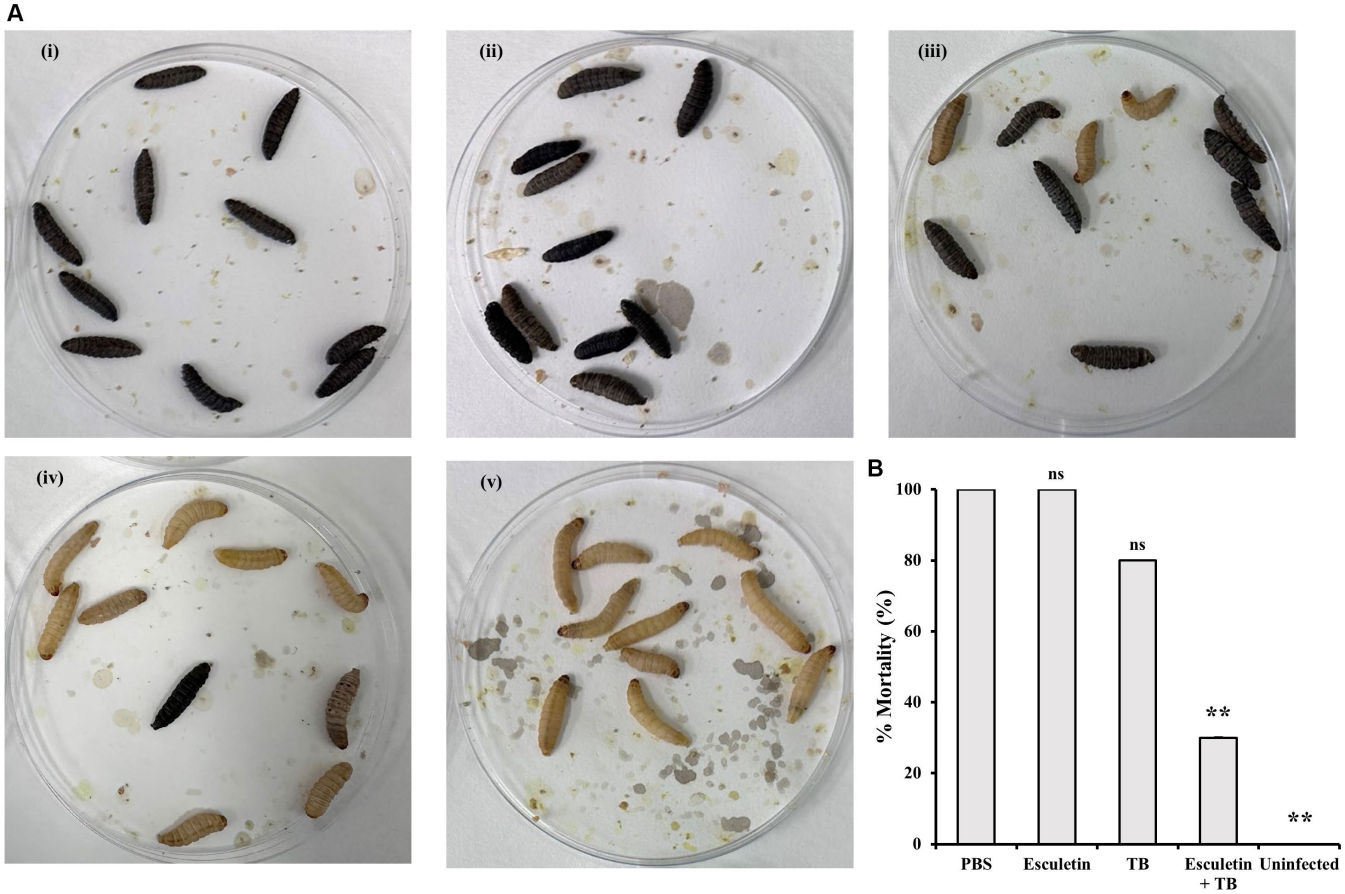
Effects of 6,7-Dihydroxycoumarin on *Galleria mellonella* larvae infection with *Pseudomonas aeruginosa*. *Galleria mellonella* larvae in infection treated with PBS **(i)**, 6,7-dihydroxycoumarin (esculetin; 200 mg/Kg; **ii**), tobramycin (1 mg/Kg; **iii**), or a combination of both **(iv)**. PBS-injected larvae without infection were the negative control **(v)**. **(A)** Representative of symptoms in *Galleria mellonella* **(B)** Percentage mortality (mean ± SD) of *Galleria mellonella* larvae. Plots show an average of three independent experiments with 10 larvae per group, with mortality monitored 16 h post treatment. ^*^p < 0.05; ^**^p < 0.01, Student’s *t*-test. Means and standard deviations from triplicate experiments are shown.

The effect of 6,7-dihydroxycoumarin on the efficacy of tobramycin (TB) treatment was further evaluated in the *Galleria mellonella* infection model. Larvae were inoculated with WT PA14 culture and subsequently treated with PBS, tobramycin (1 mg/Kg), 6,7-dihydroxycoumarin (200 mg/Kg), or a combination of both. Mortality, cuticle discoloration, and response to touch were recorded 16 h after the treatment. Control larvae infected with *P. aeruginosa* and treated with PBS exhibited 100% mortality, which was reduced to 80% by tobramycin treatment. However, the addition of 6,7-dihydroxycoumarin to tobramycin (TB) resulted in a greater reduction in mortality compared to tobramycin alone (Figure 5). Treatment with 6,7-dihydroxycoumarin alone did not show a decrease in mortality.

Altogether, the findings from both *in vitro* and *in vivo* experiments indicate that the combination of 6,7-dihydroxycoumarin with antibiotic treatment effectively reduces resistance of *P. aeruginosa.* These findings underscore the potential of 6,7-dihydroxycoumarin as a supplementary therapy to augment the efficacy of antibiotics in combatting *P. aeruginosa* infections.

## Discussion

The emergence of antibiotic resistance in bacteria is a worldwide concern, posing a significant threat to healthcare systems worldwide. In response to this urgent issue, researchers have been exploring new strategies to identify efficient anti-bacterial agents. One promising approach is targeting regulatory pathways that utilize intracellular Bis-(3′,5′) cyclic di-guanylate (c-di-GMP) as a second messenger. C-di-GMP is present in nearly all bacteria and plays a vital role in regulating various processes, including antibiotic resistance, biofilm formation, and virulence. Manipulating c-di-GMP levels through interference, which can disrupt associated signaling pathways, holds great potential to reduce biofilm formation or enhance the susceptibility of biofilm forming bacteria to antibiotics.

To investigate the effects of compounds on c-di-GMP levels and their potential as modulators of biofilm formation, antibiotic resistance, and virulence, we developed a co-culture model using bronchial epithelial cells. In this way, we hoped to mimic the complex interactions between bacterial biofilms and host cells, providing an insight into bacterial behavior and biofilm formation during the infection process.

One notable advantage of the developed screening method is its scalability. The ability to scale up the assay allows for the screening of a large number of compounds, increasing the chances of identifying potential inhibitors or modulators of c-di-GMP levels. This scalability is crucial for efficiently screening compound libraries and expanding the scope of potential therapeutic interventions. By screening a natural compound library consisting of 200 compounds, we successfully identified a potential inhibitor, 6,7-dihydroxycoumarin, which demonstrated promising effects in modulating c-di-GMP levels, biofilm formation, and virulence in *P. aeruginosa.*

6,7-dihydroxycoumarin, alternatively known as esculetin, is a naturally-occurring compound primarily sourced from the Chinese herbal medicine Fraxinus rhynchophylla Hance. This compound exhibits a wide range of pharmacological properties, encompassing antioxidant, anti-inflammatory, anticancer, antidiabetic, neuroprotective, cardiovascular protective effects, and notable antibacterial capabilities (Zhang et al., 2022).

Our experimental findings do not provide sufficient evidence to draw a definitive conclusion regarding the mechanism responsible for the esculetin-induced reduction of c-di-GMP levels in *P. aeruginosa*. It is widely acknowledged that c-di-GMP levels are intricately controlled by two enzymes with opposing actions. Specifically, c-di-GMP is synthesized from two GTP molecules by diguanylate cyclases (DGCs) and broken down into two GTP molecules via pGpG by phosphodiesterases (PDEs). The decrease in cellular c-di-GMP levels can occur through two potential routes: either by reducing the activity of DGCs containing GGDEF domains or by enhancing the activity of PDEs containing EAL and/or HD-GYP domains. It is plausible that the observed alterations in c-di-GMP levels and associated phenotypic changes induced by esculetin could be attributed to direct interference with these enzymes involved in c-di-GMP synthesis and degradation. Alternatively, it cannot be ruled out that esculetin exerts its influence on c-di-GMP levels by affecting enzymes engaged in c-di-GMP metabolism, possibly at the post-transcriptional or post-translational level. Moreover, there is the possibility that esculetin might indirectly lower c-di-GMP levels in *P. aeruginosa* through a different, as yet unidentified mechanism. To thoroughly investigate these potential mechanisms of action by esculetin, a comprehensive series of *in silico*, *in vitro*, and *in vivo* assays would be key.

Despite lack of complete understanding of the mode of action of esculetin-mediated reduction of the c-di-GMP level in *P. aeruginosa*, research into the antibacterial and anti-biofilm potential of esculetin has yielded promising results across multiple bacterium types. In one study, esculetin significantly inhibited quorum sensing (QS) and biofilm formation in *Aeromonas hydrophila*. Different concentrations of esculetin (25, 50, and 100 μg/mL) were shown to reduce the production of protease and hemolysin, impair biofilm formation, and attenuate swarming motility. The anti-biofilm activity of esculetin was confirmed using confocal laser scanning microscopy and scanning electron microscopy. Gene expression analysis revealed downregulation of genes positively related to QS and biofilm formation, and upregulation of a gene (*litR*) negatively related to biofilm formation. The alteration in gene expression aligned with the phenotypic changes, indicating the potential of esculetin as a QS inhibitor for *A. hydrophila* (Sun et al., 2021).

In addition to its impact on *A. hydrophila*, synthetic derivatives of esculetin, specifically 6,7-dihydroxycoumarin-5-carboxylates (DHCou and 4-Me-DHCou), have demonstrated effective antibiofilm properties against *Staphylococcus aureus* and *Candida albicans*. Notably, these compounds do not exhibit the cytotoxicity typically associated with the parent 6,7-dihydroxycoumarins like esculetin and 4-methylesculetin (Zscherp et al., 2023).

Esculetin has also demonstrated notable antibacterial efficacy against the phytopathogen *Ralstonia solanacearum and E. coli* O157:H7, a frequent culprit behind hemorrhagic colitis (Lee et al., 2014; Yang et al., 2016). It seems to damage the bacterial cell membrane and inhibit biofilm formation, most likely by repressing key virulence genes. These findings suggest potential applications of esculetin in antivirulence strategies for managing bacterial infections.

However, despite these promising results, the therapeutic use of esculetin is somewhat hampered by its low oral bioavailability, linked to its extensive metabolism through glucuronidation. Despite this limitation, the potential of esculetin for treating a range of conditions, from bacterial infections to chronic diseases like cancer, diabetes, and neurodegenerative disorders, is increasingly recognized.

The methodology we developed is adaptable to other infection models. Biofilm-related infections can occur in various host tissues and organs, necessitating the investigation of different contexts. By replicating the co-culture model with different host cell types, we can examine the effects of c-di-GMP modulators on biofilm formation and antibiotic susceptibility in diverse settings. This adaptability enhances the generalizability and translational potential of our findings, allowing for a broader application in combating bacterial infections.

In summary, directing efforts toward regulatory pathways that involve c-di-GMP represents a promising strategy in the fight against antibiotic-resistant bacteria. The use of a co-culture model incorporating bronchial epithelial cells enables the comprehensive study of biofilm interactions with host cells. The scalability of our screening method facilitates the screening of large compound libraries, leading to the identification of potential c-di-GMP modulators in addition to 6,7-dihydroxycoumarin. Additionally, the adaptability of the methodology to other infection models enhances its applicability in addressing biofilm-related infections in diverse host environments.

Additional research and development built upon these discoveries have the potential to make significant contributions to the creation of innovative therapeutic approaches for addressing antibiotic-resistant bacteria.

## Data availability statement

The original contributions presented in the study are included in the article/Supplementary material, further inquiries can be directed to the corresponding author.

## Ethics statement

Ethical approval was not required for the studies on humans and animals in accordance with the local legislation and institutional requirements because only commercially available established cell lines were used.

## Author contributions

YH: Data curation, Formal analysis, Investigation, Methodology, Project administration, Writing – original draft, Writing – review & editing. JW: Funding acquisition, Investigation, Project administration, Resources, Supervision, Writing – review & editing. SA: Data curation, Formal analysis, Investigation, Methodology, Project administration, Writing – original draft, Writing – review & editing, Conceptualization, Funding acquisition, Resources, Supervision.

## Footnotes

^1^ http://www.caithnessbiotechnologies.com/puretitre.html
