# Supplementary material for: Host cell-based screening assays for identification of molecules targeting Pseudomonas aeruginosa cyclic di-GMP signaling and biofilm formation
Source: Front Microbiol. 2023 Nov 15;14:1279922. doi: 10.3389/fmicb.2023.1279922 (PMC10684931; doi:10.3389/fmicb.2023.1279922)
Supplement: Supplementary file 1 [file Table_1.docx]

**Supplementary Tables**

**Supplementary Table 1. Stains and plasmids**

| **Strains/Plasmids** | **Relevant information** | **Source/Reference** |
| --- | --- | --- |
| **Strains** | | |
| *E. coli* | | |
| DH5α | *E. coli* strain for constructing recombinant plasmids | Lab stock |
| SM10 | *E. coli* strain as conjugation donor | Miller & Mekalanos, 1988 |
| pUX-BF13 | pUX-BF13 *E. coli* helper strain carrying a transposase on a pUX plasmid, amplicillin resistant | Bao *et al.,* 1991 |
| pRK600 | PRK 600 *E. coli* fertile helper strain, chloramphenicol resistant | Kessler *et al.,* 1992 |
| *P. aeruginosa* | | |
| PA14 | Wild type | Rahme *et al.,* 1995 |
| PA14-*lux* (pCdrA::*gfp^C^*) | *luxCDABE*‐tagged PA14 harboring plasmid pCdrA::*gfp* | This study |
| PA14-*gfp* | PA14 WT with chromosomal GFP tag | This study |
| **Plasmids** | | |
| pCdraA::*gfp^C^* | pUCP22Not-PcdrA-RBSII-*gfp* (Mut3)-T0-T1 | Rybtke *et al.,* 2012 |
| pUC18T-mini-Tn7T-*lux*-Gm | Suicide vector for shuttling single copies of genes directly to the chromosome via a mini-Tn7 element; *aacC1* gene encoding gentamicin resistance marker on Tn7 element; contains oriT for mobilization; P1 integron promoter driving expression of *lux*CDABE; Amp^r^ Gm^r^ | Damron *et al.,* 2013 |

**Supplementary Table 2. List of compounds with a significant effect on intracellular levels of c-di-GMP (%Inhibiting ≥ ±50%)**

| Compound name | %Inhibition_GFP_ | %Inhbition_cytotoxicity_ |
| --- | --- | --- |
| Hematoxylin | -905.8% | 53.6% |
| Alpha-Mangostin | -258.4% | 114.5% |
| Curcumin | -255.1% | -118.0% |
| Shikonin | -134.1% | 96.6% |
| Gossypol | -114.0% | 121.7% |
| Ellagic acid | -103.7% | -115.4% |
| 10-Hydroxycamptothecin | -82.3% | -136.3% |
| cis-5,8,11,14,17-Eicosapentaenoic acid | -79.5% | -186.8% |
| (-)-Scopolamine N-butyl bromide | -75.8% | -186.8% |
| (-)-Epigallocatechin gallate (EGCG) | -51.9% | -73.4% |
| 3-hydroxy myristic acid | -50.0% | -53.0% |
| Biochanin A (4-Methylgenistein) | 50.9% | -67.5% |
| Methyl anthranilate | 53.4% | 7.6% |
| 6,7-Dihydroxycoumarin | 67.7% | 92.4% |
| Tetracycline hydrochloride* | 77.8% | -2.1% |
| Doxycycline Hyclate* | 86.8% | -59.4% |
| Erythromycin* | 108.0% | -94.1% |
| Piceatannol | 110.4% | 41.1% |

*Antibiotics

**Supplementary Table 3. List of compounds with a significant ability to inhibit cytotoxicity (%Inhibition_cytotoxicity_ ≥ 50%)**

| Compound name | %Inhibition_GFP_ | %Inhbition_cytotoxicity_ |
| --- | --- | --- |
| Vanillin | 34.6% | 50.4% |
| Orotic acid (6-Carboxyuracil) | 24.4% | 51.1% |
| Luteolin | -18.0% | 51.9% |
| Phylloquinone | 21.9% | 53.1% |
| Hematoxylin | -905.8% | 53.6% |
| Chrysin | 7.3% | 54.2% |
| Xylitol | 25.6% | 57.6% |
| Solanesol | 38.5% | 59.4% |
| Hesperetin | 0.4% | 59.7% |
| Phloretin | -2.6% | 62.0% |
| Magnolol | 16.3% | 63.1% |
| Ethyl maltol | 25.0% | 63.6% |
| Tanshinone IIA | 20.7% | 63.6% |
| Abietic acid | 6.7% | 67.2% |
| Xanthone | 44.1% | 70.4% |
| Isoliquiritigenin | 14.0% | 73.6% |
| Naringin | 40.4% | 76.0% |
| Genistein | 5.8% | 77.5% |
| Kaempferol | -43.0% | 82.8% |
| Fisetin (Fustel) | -6.2% | 84.3% |
| Pterostilbene | 10.1% | 92.1% |
| 6,7-Dihydroxycoumarin | 67.7% | 92.4% |
| Sclareol | -9.3% | 95.8% |
| Shikonin | -134.1% | 96.6% |
| (+)-Usniacin | 26.1% | 106.6% |
| Parthenolide | -15.6% | 113.7% |
| Alpha-Mangostin | -258.4% | 114.5% |
| Honokiol | 7.8% | 119.7% |
| Gossypol | -114.0% | 121.7% |
| Escin | -20.0% | 122.0% |

**Reference**

Bao, Y., Lies, D. P., Fu, H., & Roberts, G. P. (1991). An improved Tn7-based system for the single-copy insertion of cloned genes into chromosomes of gram-negative bacteria. Gene, 109(1), 167–168. https://doi.org/10.1016/0378-1119(91)90604-A

Damron, F. H., McKenney, E. S., Barbier, M., Liechti, G. W., Schweizer, H. P., & Goldberg, J. B. (2013). Construction of mobilizable mini-Tn7 vectors for bioluminescent detection of gram-negative bacteria and single-copy promoter lux reporter analysis. Applied and Environmental Microbiology, 79(13), 4149–4153. https://doi.org/10.1128/AEM.00640-13/SUPPL_FILE/ZAM999104468SO2.PDF

Kessler, B., de Lorenzo, V., & Timmis, K. N. (1992). A general system to integratelacZ fusions into the chromosomes of gram-negative eubacteria: regulation of thePm promoter of theTOL plasmid studied with all controlling elements in monocopy. Molecular and General Genetics MGG 1992 233:1, 233(1), 293–301. https://doi.org/10.1007/BF00587591

Miller, V. L., & Mekalanos, J. J. (1988). A novel suicide vector and its use in construction of insertion mutations: osmoregulation of outer membrane proteins and virulence determinants in Vibrio cholerae requires toxR. Journal of Bacteriology, 170(6), 2575–2583. https://doi.org/10.1128/JB.170.6.2575-2583.1988

Rahme, L. G., Stevens, E. J., Wolfort, S. F., Shao, J., Tompkins, R. G., & Ausubel, F. M. (1995). Common virulence factors for bacterial pathogenicity in plants and animals. Science (New York, N.Y.), 268(5219), 1899–1902. https://doi.org/10.1126/SCIENCE.7604262

Rybtke, M. T., Borlee, B. R., Murakami, K., Irie, Y., Hentzer, M., Nielsen, T. E., Givskov, M., Parsek, M. R., & Tolker-Nielsen, T. (2012). Fluorescence-based reporter for gauging cyclic Di-GMP levels in Pseudomonas aeruginosa. Applied and Environmental Microbiology, 78(15), 5060–5069. https://doi.org/10.1128/AEM.00414-12/ASSET/17160CB0-7934-47F2-8768-C82F8D6D0863/ASSETS/GRAPHIC/ZAM9991034540006.JPEG
