# Supplementary figures and images for: Host cell-based screening assays for identification of molecules targeting Pseudomonas aeruginosa cyclic di-GMP signaling and biofilm formation
Source: Front Microbiol. 2023 Nov 15;14:1279922. doi: 10.3389/fmicb.2023.1279922 (PMC10684931; doi:10.3389/fmicb.2023.1279922)

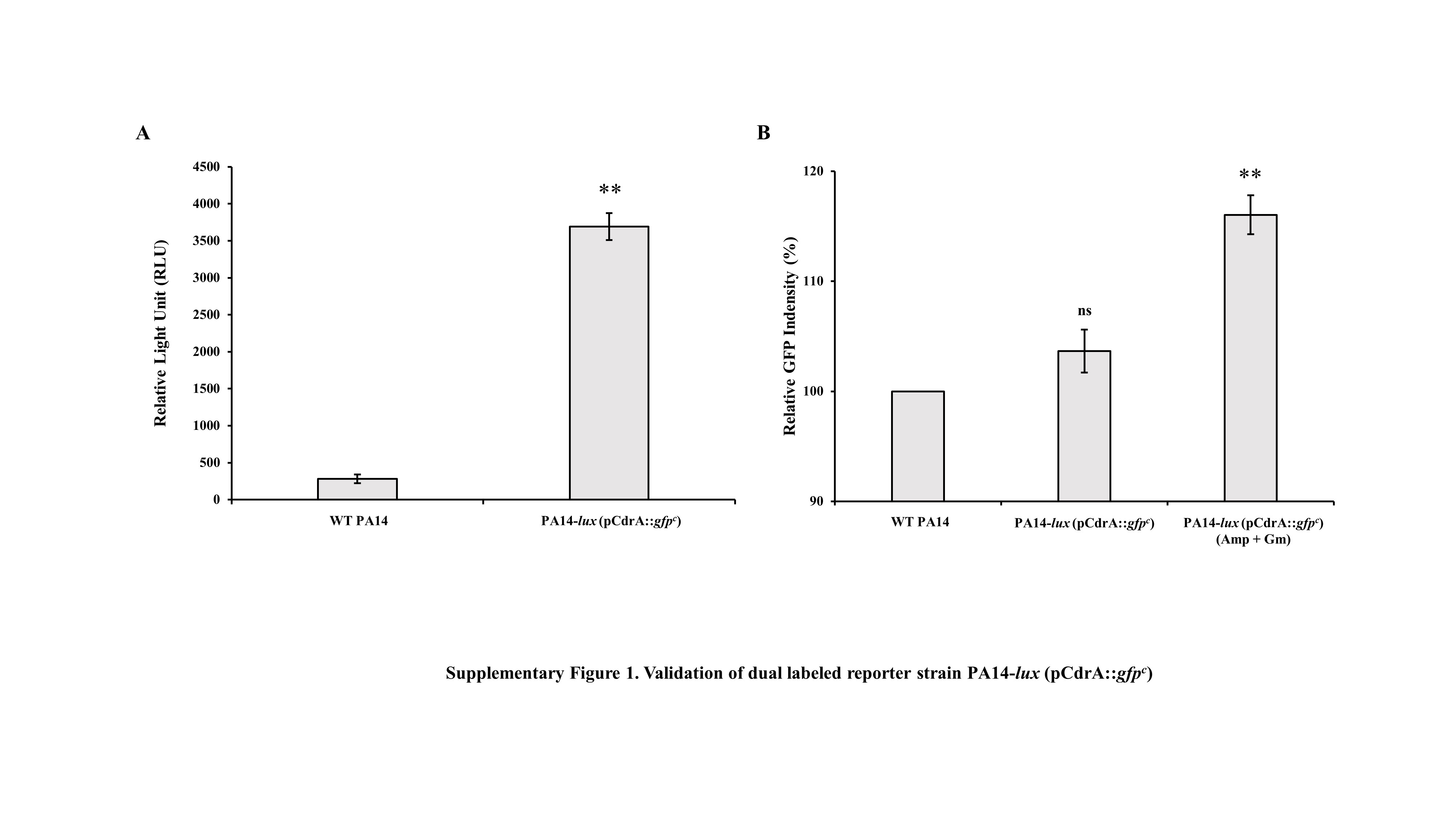

Supplement: Supplementary file 2 [file Image_1.JPEG]

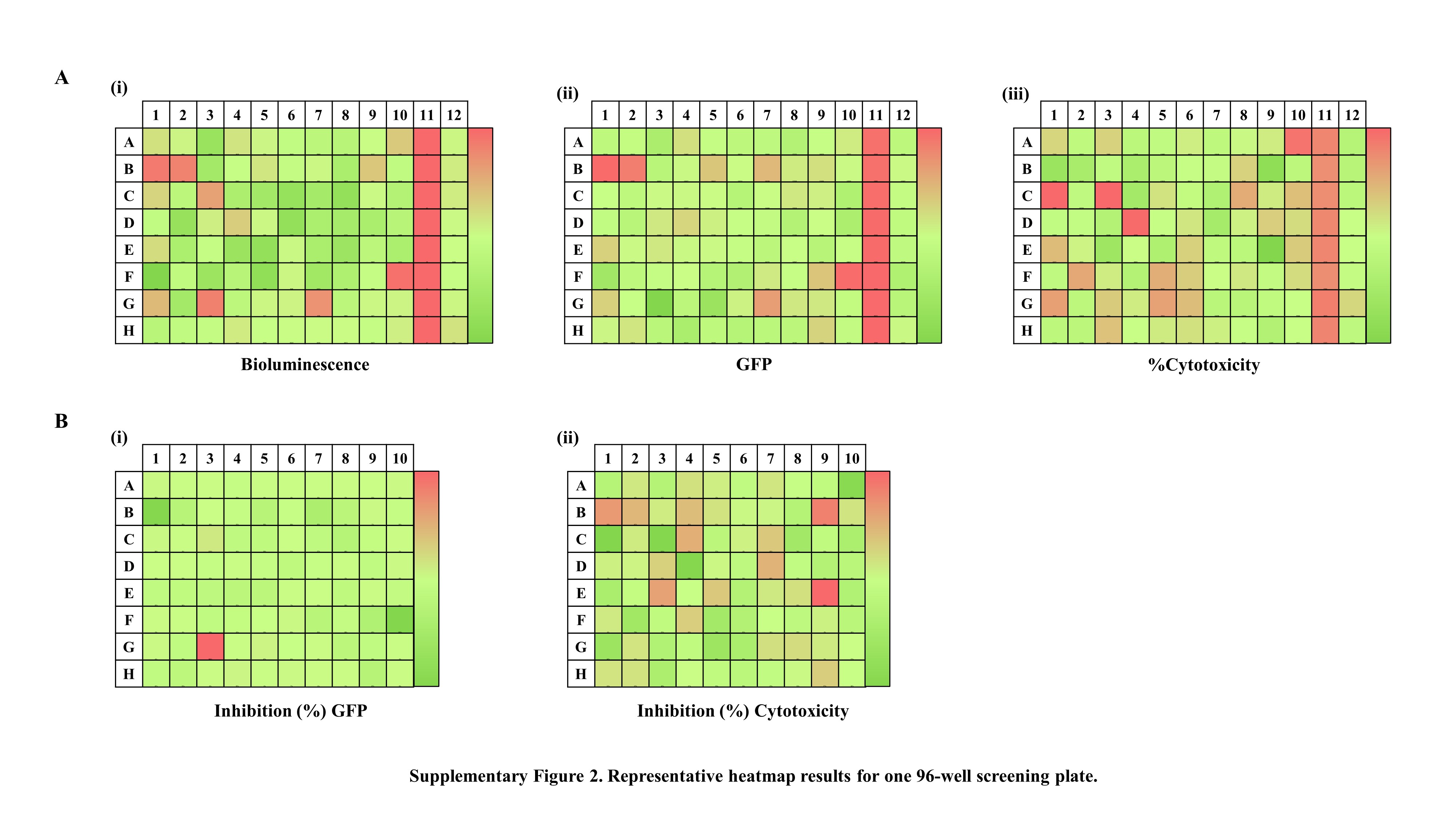

Supplement: Supplementary file 3 [file Image_2.JPEG]

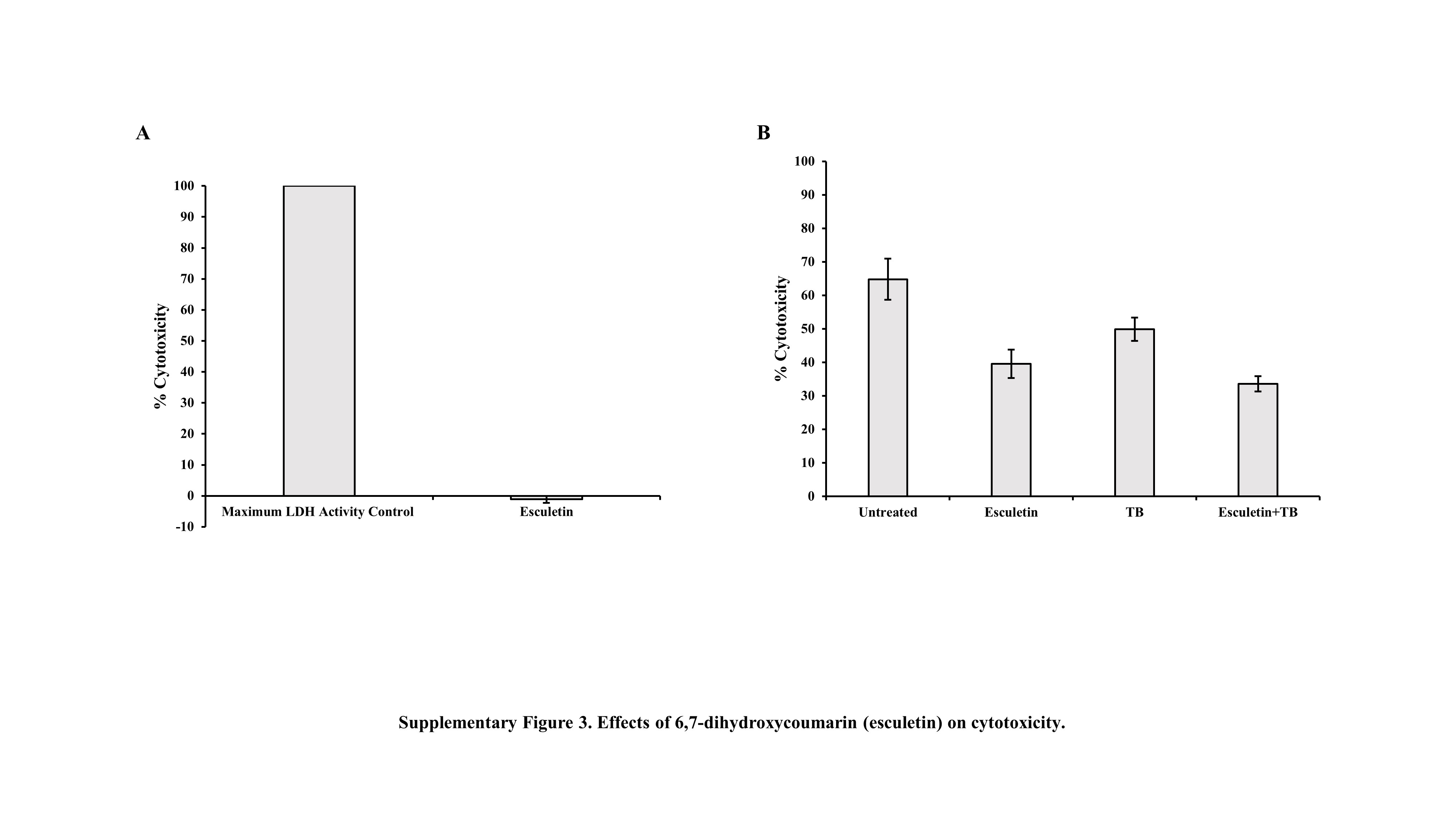

Supplement: Supplementary file 4 [file Image_3.JPEG]
